# Evaluation of a Novel Conjunctive Exploratory Navigation Interface for Consumer Health Information: A Crowdsourced Comparative Study

**DOI:** 10.2196/jmir.3111

**Published:** 2014-02-10

**Authors:** Licong Cui, Rebecca Carter, Guo-Qiang Zhang

**Affiliations:** ^1^Department of Electrical Engineering and Computer ScienceDivision of Medical InformaticsCase Western Reserve UniversityCleveland, OHUnited States; ^2^School of MedicineDepartment of Epidemiology and BiostatisticsCase Western Reserve UniversityCleveland, OHUnited States

**Keywords:** crowdsourcing, consumer health information, human computer interaction, information retrieval, search interfaces, comparative user evaluation

## Abstract

**Background:**

Numerous consumer health information websites have been developed to provide consumers access to health information. However, lookup search is insufficient for consumers to take full advantage of these rich public information resources. Exploratory search is considered a promising complementary mechanism, but its efficacy has never before been rigorously evaluated for consumer health information retrieval interfaces.

**Objective:**

This study aims to (1) introduce a novel Conjunctive Exploratory Navigation Interface (CENI) for supporting effective consumer health information retrieval and navigation, and (2) evaluate the effectiveness of CENI through a search-interface comparative evaluation using crowdsourcing with Amazon Mechanical Turk (AMT).

**Methods:**

We collected over 60,000 consumer health questions from NetWellness, one of the first consumer health websites to provide high-quality health information. We designed and developed a novel conjunctive exploratory navigation interface to explore NetWellness health questions with health topics as dynamic and searchable menus. To investigate the effectiveness of CENI, we developed a second interface with keyword-based search only. A crowdsourcing comparative study was carefully designed to compare three search modes of interest: (A) the topic-navigation-based CENI, (B) the keyword-based lookup interface, and (C) either the most commonly available lookup search interface with Google, or the resident advanced search offered by NetWellness. To compare the effectiveness of the three search modes, 9 search tasks were designed with relevant health questions from NetWellness. Each task included a rating of difficulty level and questions for validating the quality of answers. Ninety anonymous and unique AMT workers were recruited as participants.

**Results:**

Repeated-measures ANOVA analysis of the data showed the search modes A, B, and C had statistically significant differences among their levels of difficulty (*P*<.001). Wilcoxon signed-rank test (one-tailed) between A and B showed that A was significantly easier than B (*P*<.001). Paired *t* tests (one-tailed) between A and C showed A was significantly easier than C (*P*<.001). Participant responses on the preferred search modes showed that 47.8% (43/90) participants preferred A, 25.6% (23/90) preferred B, 24.4% (22/90) preferred C. Participant comments on the preferred search modes indicated that CENI was easy to use, provided better organization of health questions by topics, allowed users to narrow down to the most relevant contents quickly, and supported the exploratory navigation by non-experts or those unsure how to initiate their search.

**Conclusions:**

We presented a novel conjunctive exploratory navigation interface for consumer health information retrieval and navigation. Crowdsourcing permitted a carefully designed comparative search-interface evaluation to be completed in a timely and cost-effective manner with a relatively large number of participants recruited anonymously. Accounting for possible biases, our study has shown for the first time with crowdsourcing that the combination of exploratory navigation and lookup search is more effective than lookup search alone.

##  Introduction

The Internet has become one of the most important sources for consumers to seek health-related information. As a recent survey indicated [1], over 80% of Internet users in the United States have looked online for health information such as a specific disease or treatment, and 60% said the information found online affected decisions or actions on their own health or the way they cared for someone else.

Although a substantial amount of consumer health information is available online [2], it is not necessarily easy for general consumers to access such information. For example, a study by Berland et al [3] found that accessing health information by means of search engines (eg, Google or Yahoo) and simple search terms was not efficient. Less than a quarter of links on the search engine’s first pages of search results led to relevant content.

There are two basic information retrieval modes for accessing online health information: *lookup* and *exploratory* searches [4,5]. In lookup mode, a user comes with specific terms about a disease, medication, or other related description, enters search terms into search engines, and tries to retrieve a corresponding set of responses. For example, Berland et al [3] used lookup for the retrieval of an initial set of Web links by entering search terms such as “breast cancer”, “childhood asthma”, “depression”, and “obesity” into search engines. In exploratory mode, a user may not have a specific target, or cannot easily and effectively formulate descriptive lookup terms, and may rely on navigational menus or facets to browse and explore the content. In most cases [6-13], lookup is accompanied by exploration to help the user find a needle in a haystack. The volume of search results can be overwhelmingly large and needs to be further structured to allow relevant information to be located. For example, Mu et al [7] presented a facet-view interface complementing lookup search for effectively retrieving and navigating medical literatures in a subset of MEDLINE [14].

Navigational exploration relies on information organization to provide structures (eg, topics as menus) with which to organize a collection of contents to facilitate browsing and exploration. Consumer health questions online are often organized by categories or topics in consumer health-related Web services such as WebMD Answers [15], health category in Yahoo Answers [16], and Ask an Expert in NetWellness [17,18].

However, a common limitation of these organizational structures is that each question is assigned a single topic among a collection of available topics, even though multiple topics are related to the question. This presents a major impediment to accessing consumer health information through use of navigational exploration, such as in searching the health question repository in NetWellness.

**Figure 1:**
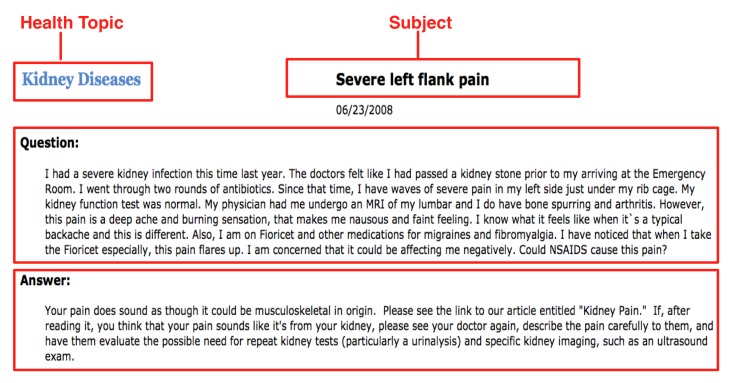
A sample consumer health question in NetWellness, consisting of four components: Health Topic, Subject, Question, and Answer.

NetWellness is a non-profit Web service providing high-quality health information. It has been in operation since 1995 with over 13 million visits per year by consumers across the world in recent years. Consumer questions in NetWellness have been answered by medical and health professional faculties at three Ohio partner universities: Case Western Reserve University, the Ohio State University, and University of Cincinnati. However, each question was assigned a single topic, thereby limiting the potential benefit of using navigational exploration. For example, although the question in Figure 1 was assigned the topic “Kidney Disease”, it can also be related to the topics “Pain Management” and “Pharmacy and Medications”. Allowing for multiple relevant topics assigned to a single question (if applicable) enables consumers to reach it through multiple pathways, thus improving the retrieval recall in navigational exploration. To categorize health questions into multiple topics, in our previous study, we used MetaMap [19] to assign CUIs (Concept Unique Identifiers) in Unified Medical Language System [20] to these questions, since CUIs allow for the handling of synonyms. The CUI tags were also used for assigning questions to one or several of the 99 predefined topics, which took the semantics of the questions’ contents into account.

In this study, we present a novel Conjunctive Exploratory Navigation Interface (CENI) for exploring NetWellness health questions with health topics as dynamic and searchable menus complementing lookup search. CENI provides a conjunctive mechanism for users to quickly drill down to relevant contents, rather than being exposed to an overwhelming number of webpages that are unlikely to be helpful.

To evaluate the effectiveness of CENI, we conducted a comparative study of search interfaces with anonymous, paid participants recruited from an online labor marketplace called Amazon Mechanical Turk (AMT) [21], a well-known and widely used crowdsourcing platform. AMT provides an attractive platform due to the relative ease of recruitment, low cost, and access to a diverse and large pool of potential participants. Crowdsourcing has been validated as a valuable method for conducting online experiments including health research [22,23], behavioral research [24,25], natural language processing [26-28], imaging analysis [29,30], drug discovery [31], and user interface evaluation [32]. Komarov et al investigated the validity of performing crowdsourcing evaluations of user interfaces, and the results provided evidence that AMT could be a productive mechanism for conducting performance evaluations of user interfaces to complement existing methodologies [32].

This study presents a novel conjunctive exploratory navigation mechanism to support consumer health information retrieval. Its efficacy is validated by conducting a crowdsourced comparative study of search interfaces for NetWellness consumer health questions.

## Methods

### Data Corpus

The data corpus used in CENI consisted of over 60,000 consumer health questions in NetWellness dated through 2012. The CENI interface relies on the underlying organization of health questions by health topics, allowing for multiple relevant topics assigned to a single question so that users can reach the question through multiple pathways. A predefined set of 99 health topics were used for tagging each question with multiple topics in our previous study.

### Conjunctive Exploratory Navigation Interface

**Figure 2:**
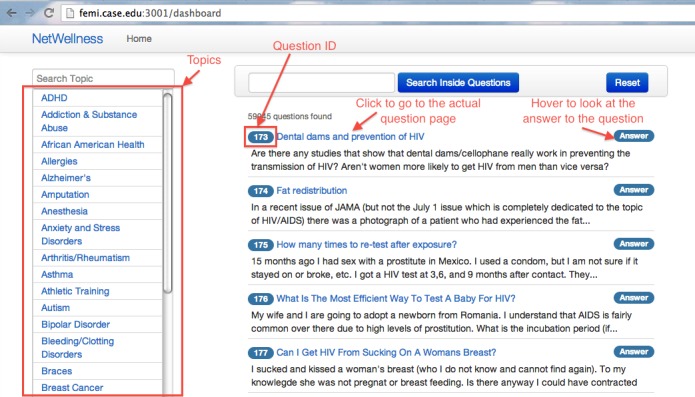
The newly developed CENI interface: a topic-based navigational exploration interface.

**Figure 3:**
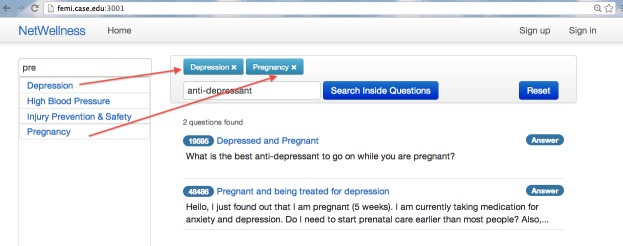
The CENI interface after selecting "Depression" and "Pregnancy" as topics, and specifying "anti-depressant" as keyword for search.

We developed the CENI interface using agile Web development with Ruby on Rails [33]. Figure 2 is a screenshot of the CENI interface, where the left column displays a list of dynamic and searchable topic menus, and the right column contains health questions. By default, all the questions are displayed if neither topic nor search keyword is specified. Figure 3 shows a sample screenshot of CENI interface after the selection of topics “Depression” and “Pregnancy”, and the user specifying “anti-depressant” as a search term to search within the returned results. In this case, the right column displays the questions tagged with all the selected topics and containing the specified keyword. The chosen topics are displayed inside the horizontal bar on the top of the right column, where the “Reset” button is used to start a new exploration by clearing the specified topics and search terms. If only a single topic is selected, all the questions tagged with the topic will be displayed. Single topic selection is equivalent to the traditional navigational exploration using static menus.

### Crowdsourcing Comparative Evaluation of Search Interfaces

#### Search Interfaces Compared

**Figure 4:**
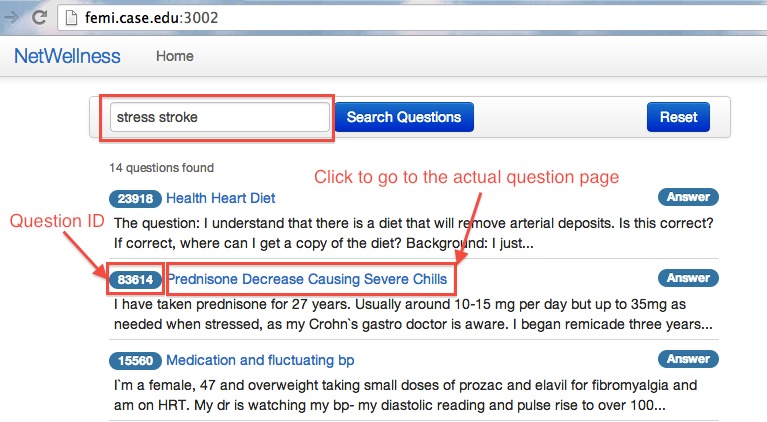
The newly developed keyword-based search interface.

**Figure 5:**
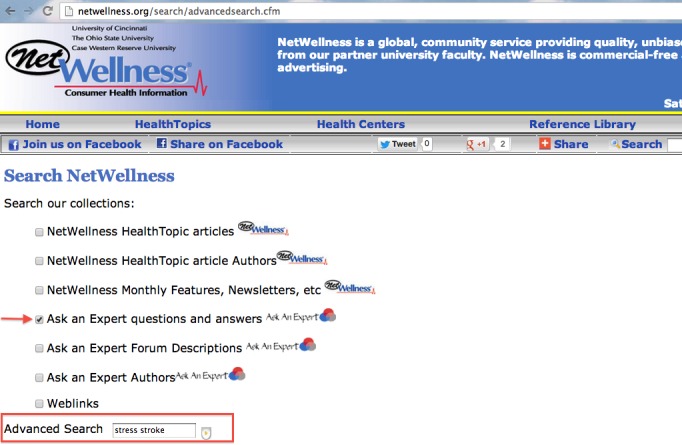
The existing advanced search feature provided in NetWellness official website.

**Figure 6:**
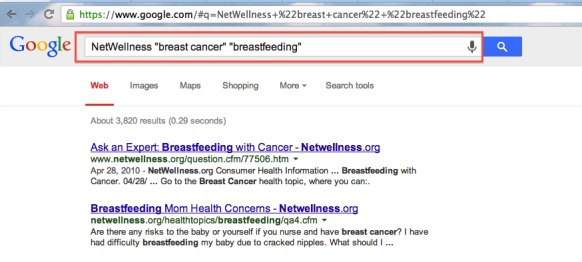
The existing Google Search interface with "NetWellness" as part of the keywords.

To evaluate the efficacy of our newly developed CENI for navigating and exploring NetWellness health questions, we performed a comparative evaluation against two other search modes using keyword-based searches. One of them is an additional newly developed interface similar to the CENI interface but with topic menus removed (Figure 4). The other is the existing keyword-based search mode either using the advanced search interface provided by NetWellness official website [34] (Figure 5) or using Google search interface [35] with “NetWellness” as part of the keywords (Figure 6). In the following descriptions, we refer to “A” (Figure 2) as the newly developed CENI interface, “B” (Figure 4) as the additional newly developed search interface with keyword-based search only, and “C” as the existing keyword-based search mode either using the advanced search in NetWellness (Figure 5) or Google Search (Figure 6).

#### Search Tasks and Rationale

To perform a comparative study of the three search modes, we designed nine search tasks (Table 1) based on relevant health questions in NetWellness. Nine tasks were selected so that we could divide the tasks into three groups to be answered by each subject using three search modes in different orders, to avoid potential order effect, in a reasonable amount of time. Intuitively, the larger the number of tasks, the less biased the study design would be. However, using more than nine tasks would entail the need to allocate a greater amount of time to the AMT workers, making it harder to recruit them (although there were sufficient financial resources available to pay the workers).

Since lookup search is a well-established area, our search tasks were designed to be mostly exploratory in nature. Therefore, each search task involved at least two health topics and had one or more related health questions. Take the search task “What might be the concerns on breastfeeding while diagnosed with breast cancer?” as an example. It involved two topics “breastfeeding” and “breast cancer”. In contrast to questions with a clear answer using a unique search string, the nine tasks were deliberately selected to demonstrate multiple navigation pathways leading to them, representing areas where the distinct search mode could potentially be optimal.

Our study is focused on this question: “Are there tasks that may definitely benefit from conjunctive search?” This has not been established before because of a lack of an interface such as CENI to support conjunctive search. We selected the nine specific tasks based on the two information retrieval modes mentioned earlier (ie, lookup and exploratory search), and they are all based on the repository of over 60,000 consumer health questions collected. Three out of the nine tasks were “lookup” in nature, which are Tasks 3, 5, and 6. The other six tasks were designed to be “exploratory” in nature. They were selected based on several criteria: (1) they should be exploratory, (2) they should be relevant in health information seeking, and (3) they should preferably have multiple associated questions in the question repository that would provide the answer (otherwise standard search would likely be sufficient).

**Table 1:** List of the nine search tasks.

Task ID	Task description
1	What are the typical vision problems associated with diabetes?
2	What are possible relationships between Alzheimer’s disease and diet?
3	Can anti-epileptic medications be taken during pregnancy?
4	What are the possible connections between smoking and erectile dysfunction?
5	Can asthma be a side effect of taking Zocor?
6	Is colon cancer an inherited disease?
7	How might Tuberculosis medication impact one’s body weight?
8	Other than prescribed medications, what other approaches may help with depression?
9	What might be the concerns on breastfeeding while diagnosed with breast cancer?

### Comparative Study Design and Procedure

We devised six surveys corresponding to six orders of search modes: ABC, ACB, BAC, BCA, CAB, CBA (see Multimedia Appendix 1 for the survey corresponding to the order ABC). To compare three search modes, we used the within-subjects design [36] with search mode as the factor, in three levels (A, B, C), which was counterbalanced appropriately to avoid order bias. In these six different surveys, the orders of the search tasks were kept the same while the orders of the search mode were permutated.

**Figure 7:**
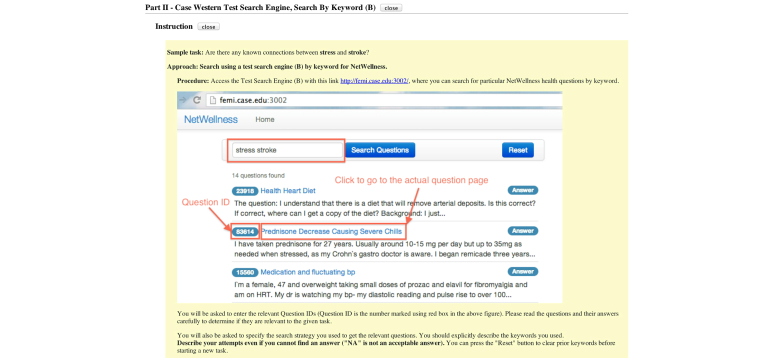
A brief instruction with screenshots for Part II.

Each survey mainly consisted of three parts, where each part had three search tasks using one type of search mode. For each part, a brief instruction with screenshots (such as Figure 7) was provided to help participants better understand how the search task could be done with a particular search mode. Each search task included a question on the rating of the level of difficulty of the task, as well as three questions that served as validation of the quality of the search task that the participant worked on. For ratings, a 9-point Likert scale was used, where “9” indicated very difficult, “5” indicated neutral, and “1” indicated very easy. The rating responses were used as primary data for the comparative statistical evaluation of three search modes.

Because of the AMT setting, this study placed greater emphasis on obtaining valid answers by the workers. To ensure the validity of the participants’ responses, the remaining three questions required participants to provide the IDs of all the relevant health questions they found, the search keywords or topics they used, and a summary of the answers given from corresponding health questions. Each participant was required to provide answers to all the four questions unless he or she made five attempts without finding any results. In that case, the attempted search keywords or topics still needed to be recorded. Responses to these three questions were used to determine the quality of the AMT worker, and if the participant’s work would be accepted or rejected. Since our objective was to evaluate the effectiveness of topic-based navigational exploration interface A, for each search task using A, we required participants to provide at least two topics from the given list for each exploration they performed. If a worker provided answers that did not match the corresponding search mode, his or her work was rejected.

During the study design phase, we considered the alternative that all answers would be accepted, correct or not, and the rate of correctness would be an evaluation criterion. We decided against this because of the paid-nature of the AMT setting: If we did not insist in obtaining correct answers (which may not be unique), we could not be sure that AMT workers were not tempted to provide arbitrary answers; then we could not guarantee that they followed the instructions carefully. A worker not following the instructions and not required to get correct answers could potentially complete the tasks in a minimum amount of time by selecting arbitrary answers, and in effect earn much higher hourly pay. This potential conflict of financial interest from the worker would have been a weakness that might have rendered the study data less useful.

Each survey also included an additional set of background questions:

1. How frequently do you use Google search? (9-point Likert scale: 1-Always, 5-Occasionally, 9-Never)
2. How often do you search for health information online? (9-point Likert scale: 1-Always, 5-Occasionally, 9-Never)
3. How would you rate your level of medical knowledge? (9-point Likert scale: 1-None, 5-Average, 9-Expert)
4. Choose your level of education. (5-point Likert scale: 1-Less than high school, 2-High school, 3-College, 4-Graduate or Higher, 5-Other)
5. Among the three health information search approaches, which one do you prefer most and why to complete the above search tasks?

These additional questions were optional but could provide information for further analysis.

For each survey, we created a separate HIT (Human Intelligence Task, the unit of paid work) on AMT. Each HIT was allocated 60 minutes for the completion of the task with US $6 compensation; 15 participants were recruited per HIT. Each was required to complete nine tasks in Table 1 (three tasks for each search mode). All participants had an approval rate of at least 85%.

Before the six surveys were given to AMT workers, a pilot survey was given to a small group of 5 AMT workers to provide feedback on (1) time needed to complete the tasks, (2) clarity of the instructions, (3) whether the nine tasks made sense, and (4) other open comments on the study design. The pilot workers found 45 minutes to be reasonable for them but suspected that more time would be beneficial because of the variations in search experiences. There were no major comments for items (2), (3), and (4). Therefore, the actual surveys for the 90 AMT workers were allotted 60 minutes.

The study involved the use of survey and assessment procedures that were obtained in such a manner that the human subjects could not be identified directly or through identifiers linked to the subjects. Therefore the study qualified as an exempt research activity by the Case Western Reserve University Institutional Review Board under the Code of Federal Regulations, 38 CFR 16.101(b) Section 3, Category 2.

## Results

### Participant Responses

Data collection for crowdsourced participant responses took place from September 18 to October 6, 2013 (a period of 18 days); 98 eligible distinct AMT workers in total were recruited. Eight workers’ responses were rejected because they were not able to complete the search tasks in the allotted time or did not follow the task instructions. As a result, the responses of 90 participants were used for the evaluation. These participants took an average of 50 minutes to complete a HIT, or survey.

Table 2 shows the numbers of participants who found relevant results using three search modes for the nine search tasks. Whether the participants found relevant results was manually validated based on their responses on the IDs of all the relevant health questions they found, the search keywords or topics they used, and a summary of the answers given in the corresponding health questions. Participants were allowed to give up a search task after five attempts were made without finding any relevant results. Tasks 3 and 8 received the least number of participants (82.2%, 74/90) who found relevant results. In sum, 90.9% (736/810) participants found relevant results: 96.3% (260/270) participants found relevant results using search mode A, 88.9% (240/270) participants found relevant results using B, and 87.4% (236/270) participants found relevant results using C.

For those who found no relevant results, manual review of the search keywords or topics provided showed that they did not devise appropriate keywords or topics. For example, five attempts made by one participant without success for Task 8 using search mode B were “depression treatments”, “depression treatment without medication”, “treating depression”, “depression and meditation”, and “depression and alternative treatments”. The following five keywords were provided by another participant for Task 8 using search mode C: “depression”, “no medication depression”, “natural depression”, “depression treatment”, and “depression approach” with no relevant results found.

**Table 2:** Number of participants who found relevant results using three search modes (A, B, C) for the nine search tasks.

Task ID	Participants who found relevant results using A, n	Participants who found relevant results using B, n	Participants who found relevant results using C, n	Participants who found relevant results, n (%)
1	30	30	30	90 (100)
2	30	24	28	82 (91.1)
3	26	24	24	74 (82.2)
4	28	29	26	83 (92.2)
5	29	30	26	85 (94.4)
6	29	30	26	85 (94.4)
7	29	24	25	78 (86.7)
8	29	20	25	74 (82.2)
9	30	29	26	85 (94.4)
Total	260 (96.3%)	240 (88.9%)	236 (87.4%)	90.9%

The information on participant responses of the first four additional questions is presented in Table 3 (see also Multimedia Appendix 2), where the numbers of participants are reported only for points less than or equal to 5. Of the 90 participants, all reported the frequency of using Google search, 89 responded to the frequency of searching health information online, 89 reported their medical knowledge levels, and 68 reported their education levels. Furthermore, 36 used Google search frequently or more (40%), and 41 occasionally used Google search (45.6%). Regarding the frequency of searching health information online, 43 reported frequently or more (47.8%), and 29 reported occasionally (32.2%). For knowledge, 79 had limited or no medical knowledge (87.8%). For the education level, 45 reported “College” education (50%), and 13 reported “Graduate or Higher” (14.4%).

Participant responses on the preference of three search interface modes are presented in Multimedia Appendix 2. All 90 participants reported their preferences; 43 participants preferred A (47.8%), 23 favored B (25.6%), 22 preferred C (24.4%), one chose A or B (1.1%), and one chose A or C (1.1%). Multimedia Appendix 3 presents the explanations of participants for preferring certain search modes.

**Table 3:** Information on participant responses of the first four additional questions (including only points on a scale less than or equal to 5).

Question	Points (≤5)	Participants, n
**Google search frequency**		
	1-Always	5
	2-Very Frequently	9
	4-Frequently	22
	5-Occasionally	41
**Health information search frequency**		
	1-Always	3
	2-Very Frequently	8
	3	22
	4-Frequently	10
	5-Occasionally	29
**Medical knowledge level**		
	1-None	44
	2	32
	3-Limited	3
	4	7
	5-Average	3
**Education level**		
	2-High school	9
	3-College	45
	4-Graduate or Higher	13
	5-Other	1

### Comparison of Three Search Modes

Participant responses for the difficulty ratings of search tasks using different search modes are also presented in Multimedia Appendix 2. For each participant, the ratings for A, B, and C were calculated by averaging the difficulty ratings of three search tasks corresponding to A, B, and C, respectively. The ratings data for each search interface mode was normally distributed.

**Figure 8:**
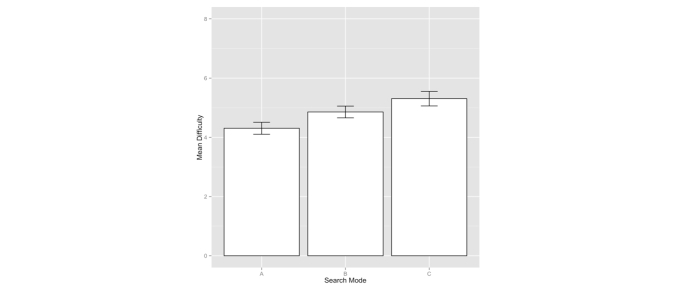
Mean difficulty ratings for search interface modes A, B, and C (1-very easy, 5-neutral, 9-very difficult) (error bar represents the standard error of the mean [SEM]; the SEMs for A, B, and C are 0.1015, 0.0999, and 0.1233, respectively).

We used the difficulty rating as a measure to compare the effectiveness of three search interface modes. Table 4 lists the average difficulty ratings of three search interface modes for the nine search tasks. For all the search tasks except Tasks 5 and 6, A was rated easier than both B and C. For all the search tasks except Tasks 2 and 8, B was rated easier than C. Figure 8 shows the mean difficulty ratings and error bars for the three search interface modes.

**Table 4:** Average difficulty ratings of three search modes (A, B, C) for the nine search tasks (1-very easy, 5-neutral, 9-very difficult).

Task ID	A	B	C
1	3.43	4	4.53
2	4.5	5.7	5.3
3	4.47	4.93	6.43
4	3.8	4.27	5.53
5	4.8	4.03	4.5
6	4.07	3.8	4.57
7	5	5.57	5.97
8	4.77	7	5.87
9	3.93	4.43	5.07
Average	4.31	4.86	5.31

Repeated-measures ANOVA analysis of the data showed statistically significant differences among the difficulty levels of A, B, and C (*P*<.001). Wilcoxon signed-rank test (one-tailed) between A and B showed that A was significantly easier than B (*P*<.001). Paired *t* tests (one-tailed) between A and C showed A was significantly easier than C (*P*<.001). Paired *t* tests (one-tailed) between B and C showed that B was significantly easier than C (*P*=.014). In sum, A (CENI) performed the best among the three.

We applied the Pearson correlation analysis to explore the effect of Google search frequency, online health information search frequency, medical knowledge, and education level on the difficulty ratings of the participants. No strong correlations were found.

According to participant comments on preferring a certain search mode most (see Multimedia Appendix 3), the most predominant reason they preferred A was that A was very easy to use and allowed users to quickly narrow down the relevant results, which is consistent with CENI’s design objective. The following are two examples of their reasons:

I liked being able to easily select multiple topics to narrow my search quickly. I found it to be the easiest search method because it decreased the number of unrelated search results.

A was much, much easier, because the problem had already divided things into these categories and I did not have to fish around at what keywords or phrases would give me the results I was looking for. I could click on any number of general topics and type in something to narrow down the search and quickly get what I was searching for.

Another reason frequently mentioned by participants was that they enjoyed the design element of the organizing information:

Explore and/or search using a test search engine by topics (A) for NetWellness. This was the easiest by far. You actually got what you wanted going this way and it was easier. I liked having the things on the side to use to begin the search.

I found A to be the best approach. Having everything in a category made it easy to narrow things down fast.

Some of the participants also noticed CENI’s benefit to less experienced users and those who are not sure exactly what to search:

Test Search Engine by Topic (A) is the most preferred by me, since it helps even a less experienced user to find and target to the topics and answers he is looking for. And for the experts, definitely, it helps to save a lot of time by optimizing the search by topics and using keywords to further filter the search.

I liked the categories. It made it easier to find what I was looking for. I would think it would be very helpful when someone is not sure exactly what search terms to use. It also seems really helpful for someone who was just diagnosed, or has questions about a disease but aren't sure what they want to know.

According to the participants who favored B (keyword-based search only), the most common reason was that it involved fewer steps to search: “I think approach B was easiest. So I prefer approach B. All I did was type a phrase I thought was relevant and it usually came up with relevant topics right away without further steps” and “I liked B the best because it was easier to use and less steps to search on it and it also gave good results. It was just clearer and less complicated than the others.”

We reviewed the keywords these participants (favoring B) provided for the search tasks, and it turned out that they were better at composing keyword phrases to acquire the most relevant results.

Among those who preferred C, the most common reason was that they were more familiar with it: “C, just because I’m more used to Google and also because I liked the way that Netwellness.org site was laid out” and “Netwellness.org/google. It was a familiar search engine to me and I found that it gave the quickest response to finding my answers.”

It is worth noting that for some participants, although they rated CENI (A) as the least difficult one, they still preferred other search modes (B or C) because they were more used to keyword-based search or Google search.

##  Discussion

### Principal Results

This study presented a novel navigational exploration interface CENI with topics as dynamic and searchable menus for consumer health information retrieval and navigation. Its conjunctive exploration mechanism allowed users to quickly drill down to the most relevant results. The efficacy of CENI was evaluated by comparing it with a similar search interface with keyword-based search only, as well as the existing search mode using Google search or NetWellness advanced search. The evaluation was conducted through crowdsourcing, a valuable method for gathering data when human participation is needed, which has been proved by many recent studies. To our knowledge, this work is the first crowdsourcing comparative evaluation of consumer health information retrieval and navigation interfaces.

We recruited 90 AMT workers as participants to work on six surveys (15 participants per survey). The surveys were carefully designed to include data quality control mechanisms and avoid order effect for comparison. The difficulty rating of search tasks using different search modes were used as a measure for the comparison. Statistical analysis showed significant differences among the difficulty rating of three search modes; CENI was significantly easier than the other two search modes. CENI was endorsed by 47.8% participants as the most preferred interface among the three search modes. Participants’ reasons for preferring CENI most were consistent with its design objective and further validated its usability.

Although CENI was implemented for organizing and exploring consumer health questions in NetWellness, it is applicable to other domains for information organization and exploration as long as the information items can be classified into multiple categories.

### Comparison With Prior Work

There were previous studies on health information retrieval and navigation [7,37]. Zeng et al [37] presented an interview and observation study in which consumers were asked to search for health information on MEDLINEplus [38,39], provided by National Library of Medicine. They analyzed the observed search sessions and determined several factors accounting for the failure of the specific searches, including confusing interface or organization of a website and information overload (too much information was retrieved). Our CENI interface presented a solution to address the potential information organization and overload challenges, by categorizing health information into one or more topics and using a conjunctive mechanism to quickly drill down to the most relevant contents.

Mu et al [7] provided a facet-view information navigation interface called SimMed for exploring literatures in a MEDLINE subset. They applied clustering technology to better organize users’ exploratory search results. The effectiveness of SimMed was evaluated by comparing to a baseline system using knowledge changes, time spent, user-system interactions and patterns, and participant preference responses. Different from SimMed, CENI organized information items by topics or categories beforehand, instead of clustering them after a user’s search. The effectiveness of CENI was measured using the quantitative difficulty level rated by the study participants and preference responses.

A unique aspect of our study is that we performed a crowdsourcing evaluation to compare search interfaces in a timely and cost-effective manner with a relatively large number of study participants recruited anonymously, rather than the traditional participatory recruitment in [7] and [37]. As far as we know, this is the first study of this kind performing direct comparison on consumer health information search interfaces.

### Limitations of Using AMT

The first limitation of AMT is that the worker population has relatively higher levels of education (college or above) and is more tech-savvy compared to the general population, and therefore may not be representative [23,30]. However, large crowds like AMT workers are certainly more representative and cost-effective than the convenience samples in traditional participatory studies, which may consist of fewer participants due to time and labor constraints [7,30,40]. In addition, crowdsourcing provides access to a population that would not be readily available through traditional methods [23]. They are also more representative of the population that is familiar with and uses the Internet as an information source.

The second limitation for AMT is that it is harder to track time spent on individual tasks. AMT can keep track only of HIT time, and not subtasks within a HIT. Breaking down each search mode as a separate HIT would have the advantage of being able to automatically track time for each mode as a HIT level functionality provided by AMT. However, it would have two potential disadvantages: (1) it would not be a paired-design (which has more statistical power), since the same AMT worker would not have worked on all three modes, and (2) there would have been 18, as opposed to 6 HITs to manage, which would be substantial overhead for managing the study.

The third limitation of AMT is that it does not currently allow weblog information to be provided to track user-system interactions and patterns. We considered using weblogs for this purpose but did not pursue it in the end for two reasons: (1) it would have introduced an additional layer of complexity in an already complex survey (compared to typical AMT surveys), and (2) although we could have tracked IP addresses and weblog patterns for the site hosting CENI, obtaining weblogs from NetWellness’ official site and Google in order to identify the specific group of AMT workers who used their search facilities would be prohibitive due to the anonymous nature of AMT workers and the need to obtain data that are neither easily identifiable nor under our control.

The fourth limitation of AMT is a predefined time limit to complete the survey. An important factor enabling measuring user-system interactions and patterns mentioned in [7] is that there was no time limitation for participants to complete their search tasks. However, for each AMT HIT, a total allocated time must be specified beforehand.

A potential fifth limitation could be that an AMT worker might be biased towards the new interface to be evaluated in an effort to please the study designer and facilitate approval of their work. Our design implemented cross-validation questions and required intensity of focus to work on all nine search tasks, thereby minimizing this possibility. In fact, some evaluators still preferred C while in fact their rating for A is the easiest, indicating no intention to please the study designer.

### Limitations of Study Design

The first limitation of our study was its use of the difficulty rating on individual tasks and modes as the major criterion. Tracking the amount of time an AMT worker took for each individual search mode would have been a useful source of information, as indicated in [7]. However, due to the limitation of AMT, it was less feasible to obtain this information without incurring other compromises in study design. We do suspect that less time would be correlated to lower levels of difficulty rating, however. And our overall preference rating and open commentary provided insight that this limitation did not affect the validity of the results.

The second potential limitation was that we selected nine search tasks, instead of a random selection of a larger number of tasks. Admittedly, more and randomly selected tasks would provide more statistical power. However, given the focused goal of the study to demonstrate the potential value of exploratory navigation using interfaces like CENI, the careful selection of search tasks was necessary, since our goal was not to show that CENI always performed better on all search tasks, lookup or exploratory.

The third potential limitation was that our CENI interface used the 99 existing NetWellness topics instead of developing an independent set of topics for tagging the questions. Our rationale was that a new set of topics dedicated for CENI would be an unfair advantage and would have introduced a major compounding factor, since the interface we compared against involved the exiting NetWellness resident search interface using the 99 topics. Also, we used the automatic multi-topic assignment results obtained in our previous study, where the precision and recall were 0.849 and 0.774 respectively. Nonetheless, this apparently did not affect our evaluation of the effectiveness of CENI, since it still outperformed the other two search modes. The correct and complete assignment of topics to questions would have definitely improved the effectiveness of the CENI interface more.

### Conclusions

We presented a novel navigational exploration interface for consumer health information retrieval and navigation. With topics presented as dynamic and searchable menus, CENI’s conjunctive exploration mechanism allowed users to quickly drill down to the most relevant results in the most effective way.

AMT crowdsourcing allowed us to perform a comparative search interface evaluation in a timely and cost-effective manner with a relatively large number of study participants recruited anonymously. Our careful study, accounting for possible biases with cross-validation for results, has confirmed that CENI does enhance consumer health information access.

## Multimedia Appendix 1

Example of Web form used for performing evaluation of search interfaces in AMT.

## Multimedia Appendix 2

Participant responses on the difficulty ratings of search tasks using different search modes.

## Multimedia Appendix 3

Participant explanations for preferring a certain search interface mode most.

## Abbreviations

AMT: Amazon Mechanical Turk
CENI: Conjunctive Exploratory Navigation Interface
CUI: concept unique identifier
HIT: human intelligence task
