# Supplementary material for: Evaluation of a Novel Conjunctive Exploratory Navigation Interface for Consumer Health Information: A Crowdsourced Comparative Study
Source: J Med Internet Res. 2014 Feb 10;16(2):e45. doi: 10.2196/jmir.3111 (PMC3936301; doi:10.2196/jmir.3111)
Supplement: Supplementary file 1 [file jmir_v16i2e45_app1.html]

### Case Western Reserve University Study: Comparison of Search and Exploration Mechanisms for a Health Question Database

**If you have worked on any similar HIT with title "Consumer Health Question Search" and requester "smartcong" before, 
please don't accept this HIT and stop here.**

**Overview**  close

The purpose of this task is to evaluate effectiveness of a
health and wellness search engine and its related user interfaces. 
This assignment compares three methods of finding consumer health questions online
through an established 
search engine NetWellness (or NW) and Google, or two optimized test search engines developed at Case Western Reserve University.

Workers are asked to:

- (A) Search the NW health question database by **topic** with a test search engine (femi.case.edu:3001)
- (B) Search the NW health question database by **keyword** with a test search engine (femi.case.edu:3002)
- (C) Use netwellness.org and/or google.com (add the keyword "netwellness" to search strings) to directly search for particular health questions

Your work has four parts. The first three parts (Part I, Part II, and Part III)
ask workers to provide specific answers  based on NW consumer health questions
using methods A, B and C above, respectively. The method used for each part may
follow a different order, such as using B for Part I, A for Part II, and C for Part III, 
instead
of merely A for Part I, B for Part II, and C for Part III.

The first three parts all begin with a visual example demonstrating
how to execute the particular task. 
Keep in mind that the purpose is not to get the answer in the quickest way possible - correctness is important!
You are required to find as many relevant
questions as possible for a given task. 
 Please follow the instructions carefully and note the following:

- The instructions for each component can be expanded or collapsed by clicking "open" or "close"
- All answers should be strictly based on information provided in NW questions.
  Evidence must be provided as NW question IDs  (unless you have attempted five times and could not find all answers)
- Be sure to rate the difficulty level after completing each task

The last part, Additional Info, provides info about
your experience with finding consumer health information online.

Part I - Case Western Test Search Engine, Search by Topic (A)  open

**Instruction**  close

**Sample task:** Can anti-depressant medications be taken during pregnancy?

**Approach: Explore and/or search using a test search engine by topics (A) for NetWellness.**

**Procedure:** Go to the Test Search Engine (A) through this link http://femi.case.edu:3001/, where the right column contains health and wellness-related questions, and the left column displays their corresponding list of topics.
**Before beginning the tasks, familiarize yourself with the topics on the left column of the screen (see figure below).**

For the 3 tasks in this part, **find the relevant questions by selecting at least two topics on the left column of the screen first. "Search Inside Questions" can only be used after you have chosen relevant topics.**
You can look up the topic list using the scroll bar or type what you have in mind in the
"Search Topic" box (e.g. "depression"). After choosing at least two topics, you can type your keywords and search among the questions determined by your chosen topics.

You will be asked to enter the relevant Question IDs (Question ID is the number marked using red box in the above figures). Please read the questions and their answers carefully to determine if they are relevant to the given task. You should explicitly describe the topics and/or keywords you used. For example, **Topics: Pregnancy, Depression; Search keyword: anti-depressant**.
**Describe your attempts even if you cannot find an answer ("NA" is not an acceptable answer).**
You can press the "Reset" button to clear prior topics or keywords before starting a
new task.

**REMINDER: You must choose at *least* two topics per task for Part II, otherwise your HIT will not be accepted.**

**Part I Tasks**

1. **What are the typical vision problems associated with diabetes?**

   Enter all relevant question IDs you found (there should be at least two, but skip this after you made five attempts without any results):

   Please explicitly describe the topics and/or search keyword(s) you used (even if no relevant questions were found):

   If you have found relevant question(s), what are the problems mentioned?

   How would you rate the level of difficulty of this task?
   --
   1-Very Easy
   2
   3-Easy
   4
   5-Neutral
   6
   7-Difficult
   8
   9-Very Difficult
2. **What are possible relationships between Alzheimer's disease and diet?**

   Enter all relevant question IDs you found (skip this after you made five attempts without any results):

   Please explicitly describe the topics and/or search keyword(s) you used (even if no relevant questions were found):

   If you have found relevant question(s), what are the possible relationships?

   How would you rate the level of difficulty of this task?
   --
   1-Very Easy
   2
   3-Easy
   4
   5-Neutral
   6
   7-Difficult
   8
   9-Very Difficult
3. **Can anti-epileptic medications be taken during pregnancy?**

   Enter all relevant question IDs you found (there should be at least two, but skip this after you made five attempts without any results):

   Please explicitly describe the topics and/or search keyword(s) you used (even if no relevant questions were found):

   If you have found relevant question(s), what are the medications mentioned?

   How would you rate the level of difficulty of this task?
   --
   1-Very Easy
   2
   3-Easy
   4
   5-Neutral
   6
   7-Difficult
   8
   9-Very Difficult

Part II - Case Western Test Search Engine, Search By Keyword (B)  open

**Instruction**  close

**Sample task:** Are there any known connections between **stress** and **stroke**?

**Approach: Search using a test search engine (B) by keyword for NetWellness.**

**Procedure:** Access the Test Search Engine (B) with this link http://femi.case.edu:3002/, where you can search for particular NetWellness health questions by keyword.

You will be asked to enter the relevant Question IDs (Question ID is the number marked using red box in the above figure). Please read the questions and their answers carefully to determine if they are relevant to the given task.

You will also be asked to specify the search strategy you used to get the relevant questions. You should explicitly describe the keywords you used.  
**Describe your attempts even if you cannot find an answer ("NA" is not an acceptable answer).**
You can press the "Reset" button to clear prior keywords before starting a new task.

**Part II Tasks**

1. **What are the possible connections between smoking and erectile dysfunction?**

   Enter all relevant question IDs (there should be at least two, but skip this after you made five attempts without any results):

   Please explicitly describe the search keyword(s) you used (even if no relevant questions were found):

   If you have found relevant question(s), what are the possible connections between the two?

   How would you rate the level of difficulty of this task?
   --
   1-Very Easy
   2
   3-Easy
   4
   5-Neutral
   6
   7-Difficult
   8
   9-Very Difficult
2. **Can asthma be a side effect of taking Zocor?**

   Enter all relevant question IDs you found (there should be at least two, but skip this after you made five attempts without any results):

   Please explicitly describe the search keyword(s) you used (even if no relevant questions were found):

   If you have found relevant question(s), what are the conclusions?

   How would you rate the level of difficulty of this task?
   --
   1-Very Easy
   2
   3-Easy
   4
   5-Neutral
   6
   7-Difficult
   8
   9-Very Difficult
3. **Is colon cancer an inherited disease?**

   Enter all relevant question IDs you found:

   Please explicitly describe the search keyword(s) you used (even if no relevant questions were found):

   If you have found relevant question(s), what are the conclusions?

   How would you rate the level of difficulty of this task?
   --
   1-Very Easy
   2
   3-Easy
   4
   5-Neutral
   6
   7-Difficult
   8
   9-Very Difficult

Part III - Netwellness.Org/Google.com (C)  open

**Instruction**  close

**Sample task:** Are there any known connections between **stress** and **stroke**?

**Approach: Search directly using netwellness.org and/or google.com, with "netwellness" as part of the search keyword if latter (C).**

**Procedure:** You can directly search within the NetWellness website http://netwellness.org/. You can type what you have in mind in the "Search" box (red box 1), or use "Advanced" search (red box 2), or navigate to relevant topics using "Health Topic" (red box 3).

If you are using the advanced search (red box 2) page http://netwellness.org/search/advancedsearch.cfm, choose "Ask an Expert questions and answers" (red arrow) and type what you have in mind for the given task in the "Advanced Search" box (e.g., "stress stroke"):

You can also use Google search by including "netwellness" as one of your keywords as well as your choice of a health topic (e.g., "netwellness stress stroke"). **Note that the answers you provided should be based on the NetWellness health questions (not from other website found in google).**

You can use any combination of the above mentioned methods to locate NetWellness health questions. But if you could not find relevant questions using Google search, **make sure that you search inside NetWellness website.**

You will be asked to enter the relevant Question IDs (Question ID is the number contained in the URL of your web browser as marked using red box in the following figure). Please read the questions and their answers carefully to determine if they are relevant to the given task.

You will also be asked to specify the search strategy you
used to get the relevant questions. You should explicitly describe the methods(s) and search keyword(s) you used. For example, Google search: netwellness stress stroke; NetWellness advanced search: stress stroke; NetWellness health topic: Anxiety and Stress Disorders.**Describe your attempts even if you cannot find an answer ("NA" is not an acceptable answer).**

**Part III Tasks**

1. **How might Tuberculosis medication impact one's body weight?**

   Enter all relevant question IDs you found (skip this after you made five attempts without any results):

   Please explicitly describe the method(s) and search keyword(s) you used (even if no relevant questions were found):

   If you have found relevant question(s), what is the impact?

   How would you rate the level of difficulty of this task?
   --
   1-Very Easy
   2
   3-Easy
   4
   5-Neutral
   6
   7-Difficult
   8
   9-Very Difficult
2. **Other than prescribed medications, what other approaches may help with depression?**

   Enter all relevant question IDs you found (skip this after you made five attempts without any results):

   Please explicitly describe the method(s) and search keyword(s) you used (even if no relevant questions were found):

   What are the other approaches mentioned in the questions?

   How would you rate the level of difficulty of this task?
   --
   1-Very Easy
   2
   3-Easy
   4
   5-Neutral
   6
   7-Difficult
   8
   9-Very Difficult
3. **What might be the concerns on breast feeding while diagnosed with breast cancer?**

   Enter all relevant question IDs (there should be at least two, but skip this after you made five attempts without any results):

   Please explicitly describe the method(s) and search keyword(s) you used (even if no relevant questions were found):

   What are the concerns mentioned in the questions?

   How would you rate the level of difficulty of this task?
   --
   1-Very Easy
   2
   3-Easy
   4
   5-Neutral
   6
   7-Difficult
   8
   9-Very Difficult

Additional Info   close

1. How frequently do you use Google search?
   --
   1-Always
   2-Very Frequently
   3
   4-Frequently
   5-Occasionally
   6-Rarely
   7
   8-Very Rarely
   9-Never
2. How often do you search for health information online?
   --
   1-Always
   2-Very Frequently
   3
   4-Frequently
   5-Occasionally
   6-Rarely
   7
   8-Very Rarely
   9-Never
3. How would you rate your level of medical knowledge?
   --
   1-none
   2
   3-limited
   4
   5-average
   6
   7-good
   8
   9-expert
4. Choose your level of education:
   --
   1-Less than high school
   2-High school
   3-College
   4-Graduate or Higher
   5-Other
5. Among the three health information search approaches: 
   **Test Search Engine by Topic (A), Test Search Engine by Keyword (B), or Netwellness.org/Google** (C), 
   **which one** do you prefer most and **why** to complete the above search tasks?

     
   Please make sure that all the ratings for the difficulty level of each task are completed, otherwise your work will not be accepted.   
    THANK YOU!
